# Transcriptional repression of *PTEN* in neural cells using CRISPR/dCas9 epigenetic editing

**DOI:** 10.1038/s41598-020-68257-y

**Published:** 2020-07-09

**Authors:** C. Moses, S. I. Hodgetts, F. Nugent, G. Ben-Ary, K. K. Park, P. Blancafort, A. R. Harvey

**Affiliations:** 1 School of Human Sciences, Faculty of Science, The University of Western Australia, 35 Stirling Highway, Perth, WA 6009 Australia; 2 Cancer Epigenetics Laboratory, The Harry Perkins Institute of Medical Research, 6 Verdun Street, Nedlands, WA 6009 Australia; 3 Perron Institute for Neurological and Translational Science, 8 Verdun Street, Nedlands, WA 6009 Australia; 4 School of Molecular Sciences, Faculty of Science, The University of Western Australia, 35 Stirling Highway, Perth, WA 6009 Australia; 5 Department of Neurological Surgery, Miami Project to Cure Paralysis, University of Miami Miller School of Medicine, Miami, FL 33136 USA; 6 Greehey Children’s Cancer Research Institute, UT Health San Antonio, 8403 Floyd Curl Drive, San Antonio, TX 78229 USA

**Keywords:** Genetic engineering, Gene therapy, Nucleic-acid therapeutics, Epigenetics, RNAi, Regeneration and repair in the nervous system

## Abstract

After damage to the adult mammalian central nervous system (CNS), surviving neurons have limited capacity to regenerate and restore functional connectivity. Conditional genetic deletion of *PTEN* results in robust CNS axon regrowth, while *PTEN* repression with short hairpin RNA (shRNA) improves regeneration but to a lesser extent, likely due to suboptimal *PTEN* mRNA knockdown using this approach. Here we employed the CRISPR/dCas9 system to repress *PTEN* transcription in neural cells. We targeted the *PTEN* proximal promoter and 5′ untranslated region with dCas9 fused to the repressor protein Krüppel-associated box (KRAB). dCas9-KRAB delivered in a lentiviral vector with one CRISPR guide RNA (gRNA) achieved potent and specific *PTEN* repression in human cell line models and neural cells derived from human iPSCs, and induced histone (H)3 methylation and deacetylation at the *PTEN* promoter. The dCas9-KRAB system outperformed a combination of four shRNAs targeting the *PTEN* transcript, a construct previously used in CNS injury models. The CRISPR system also worked more effectively than shRNAs for *Pten* repression in rat neural crest-derived PC-12 cells, and enhanced neurite outgrowth after nerve growth factor stimulation. *PTEN* silencing with CRISPR/dCas9 epigenetic editing may provide a new option for promoting axon regeneration and functional recovery after CNS trauma.

## Introduction

The devastating consequences of physical trauma, stroke or chronic neurodegenerative disease on central nervous system (CNS) function are largely due to the lack of effective repair mechanisms and the inability to regenerate neural circuitry after damage. Inflammatory changes, breakdown of CNS myelin, glial scar tissue and loss of extracellular guidance cues all contribute to an inhibitory environment that negatively impacts on axon regeneration^1–3^. Perturbation of these extracellular inhibitory factors, along with exogenous administration of supportive neurotrophic factors, can improve axon regeneration following injury to some extent^4,5^. However, mature CNS neurons also have intrinsic limitations in their responsiveness to environmental trophic factors and associated capacity for target-independent survival and axon extension^6–8^.

**Figure 1:**
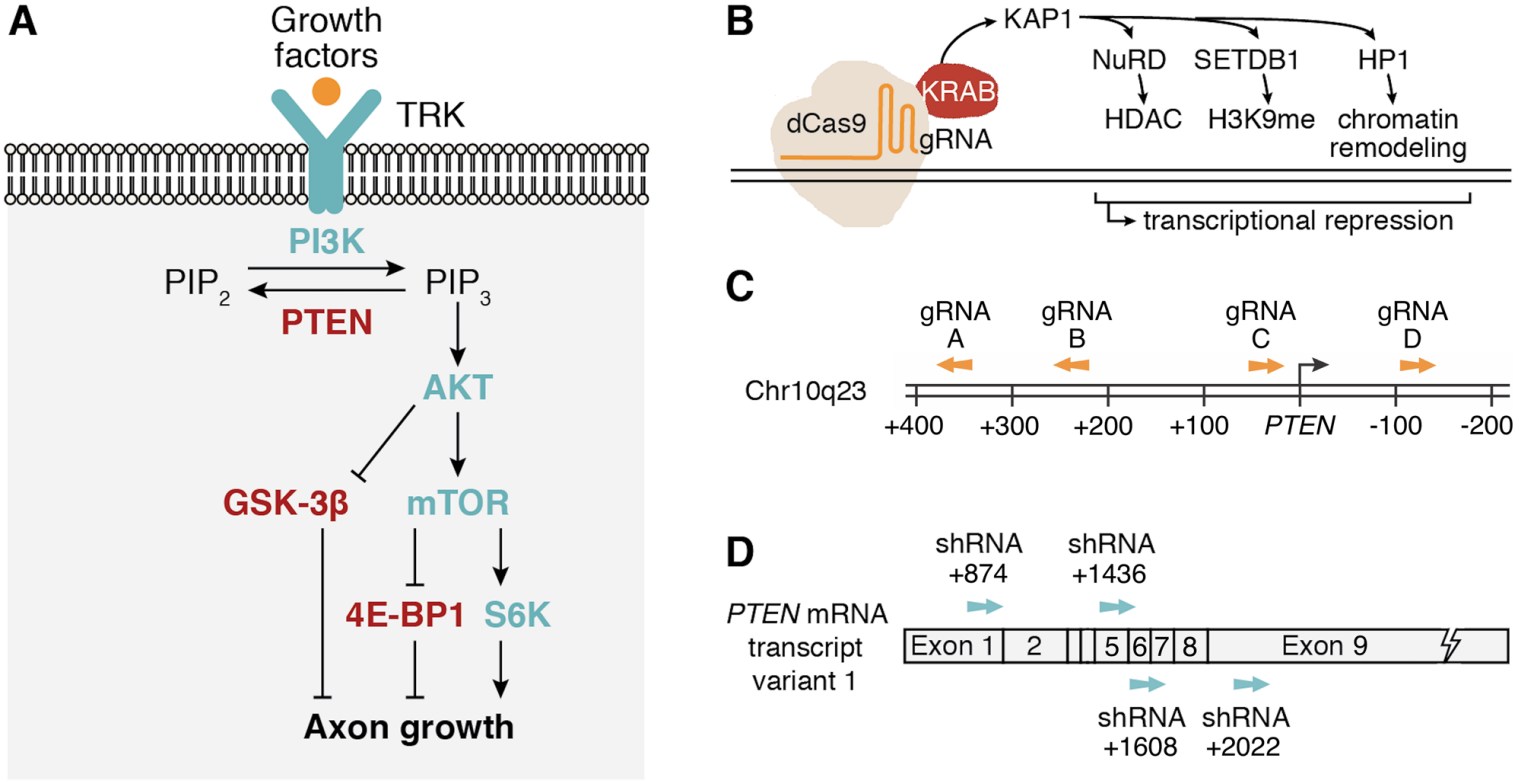
Design of CRISPR and shRNA systems for *PTEN* repression. (**A**) Intracellular signaling pathways regulating axon regeneration after CNS injury. Growth factors activate tyrosine receptor kinases (TRK), causing PI3K to convert PIP_2_ to the second messenger PIP_3_. PIP_3_ accumulation results in activation of the AKT/mTOR pathway and modulation of downstream signaling proteins GSK-β, 4E-BP1 and S6K to promote axon growth. PTEN inhibits this pathway by converting PIP_3_ to PIP_2_, which counteracts PI3K activity, reducing axon growth. (**B**) *S. pyogenes* dCas9 with C-terminal fusion of the KRAB repressor domain is directed to the DNA target site by the gRNA. KRAB recruits KAP1, which in turn engages the nuclease remodeling and deacetylase (NuRD) complex for histone deacetylation (HDAC), histone-lysine *N*-methyltransferase SETDB1 for histone methylation (H3K9me), and heterochromatin protein 1 (HP1) for chromatin remodeling. Together these effectors promote heterochromatin formation and transcriptional silencing. (**C**) Location of gRNA target sites within the *PTEN* proximal promoter and 5′ untranslated region (UTR). Numbering refers to the distance in DNA base pairs upstream or downstream of the *PTEN* transcription start site (TSS) (NM_000314.8). Arrows indicate whether the gRNA targets the forward or reverse DNA strand. (**D**) Location of shRNA target sites in the *PTEN* transcript. Exon numbering refers to the number of nucleotides downstream of the TSS in *PTEN* mRNA transcript variant 1 (NM_000314.8), however these shRNAs target all annotated *PTEN* transcript variants.

Various transcription factors and intracellular signaling proteins have been implicated in this loss of intrinsic growth ability in CNS neurons^8–11^. In particular, the phosphoinositide 3-kinase (PI3K)/mammalian target of rapamycin (mTOR) pathway plays a crucial role in influencing cell survival, protein synthesis and cytoskeleton formation necessary for axon extension after injury (Fig. 1A)^12–16^. Cre-driven deletion of the primary antagonist of the PI3K/mTOR pathway, *PTEN* (*phosphatase and tensin homolog*) produced marked improvements in axon regeneration after CNS injury in floxed mice^17–22^. Conditional genetic deletion of *PTEN* in CNS neurons improved neuronal survival and long-distance regeneration in both retinal ganglion cells^17,18^ and corticospinal neurons^19^. Importantly, axon regeneration was significantly improved when *PTEN* deletion was performed shortly after spinal cord injury, and also up to 1 year later^20,21^. *PTEN* repression is thus a promising strategy for improving axon regeneration in the damaged CNS.

As conditional genetic deletion of *PTEN* using Cre-Lox recombination is not applicable clinically, several groups have designed RNA interference strategies to knock down the *PTEN* transcript, which may be more amenable to clinical translation^23–26^. shRNAs targeting *PTEN* have been delivered to the injured spinal cord or optic nerve by adeno-associated virus (AAV), resulting in some regeneration of damaged axons which formed synapses in target regions distal to the injury site^25,26^. However, in these studies *PTEN* shRNA showed only modest levels of knockdown of *PTEN*, and the extent of axon regeneration was not as significant as with genetic *PTEN* deletion, likely due to residual *PTEN* expression^25,26^. A method that could repress *PTEN* to a similar extent as genetic deletion could provide a promising translational option for improving the response to CNS injury.

We were interested in whether epigenetic editing to repress *PTEN* at the transcriptional level could provide a more effective alternative to shRNA *PTEN* inhibition. Recently, the mechanisms underlying the Clustered Regularly Interspaced Short Palindromic Repeats (CRISPR)/CRISPR-associated protein 9 (Cas9) system of *Streptococcus pyogenes* were elucidated and subsequently adapted as a novel programmable tool for gene editing in mammalian cells^27–29^. The Cas9 endonuclease is directed to a target genomic location by a complementary guide RNA (gRNA) molecule, where it cleaves the DNA strand. Cas9-induced DNA double-strand breaks can be exploited for gene knockout. However, we favored a strategy for reversible repression of *PTEN*, which would circumvent long-term side effects imposed by permanent *PTEN* knockout^30,31^. The CRISPR system has been adapted for transcriptional activation, repression, and epigenetic editing by mutations to the catalytic domains of Cas9 to form a ‘dead’ Cas9 (dCas9) protein, which binds the DNA target specified by the gRNA without initiating a double-strand break. dCas9 has been fused to a variety of proteins, termed effector domains, that influence transcription or edit epigenetic marks when directed to regulatory regions by the gRNA^32^.

We employed dCas9 to direct the transcriptional repressor Krüppel-associated box (KRAB) to the *PTEN* transcription start site. KRAB originates from naturally occurring eukaryotic transcription factors, and has previously been fused to dCas9 and targeted to regulatory regions to achieve potent transcriptional repression^33–35^. KRAB recruits KRAB-associated protein 1 (KAP1), thereby engaging histone deacetylases (HDACs) and histone methyltransferases (HMTs) to promote heterochromatin formation (Fig. 1B)^36–41^. dCas9-KRAB fusion proteins reduce H3K9 and H3K27 acetylation, increase H3K9 and H3K27 trimethylation, and reduce chromatin accessibility at targeted regions^42–44^, and induce transcriptional silencing of target genes when directed to proximal promoters and enhancers^44–46^.

We sought to investigate whether *PTEN* expression could be effectively silenced in CNS neurons by epigenetic editing using CRISPR/dCas9-KRAB. The repression of *PTEN* without permanent gene knockout is a key advantage of this approach, as sustained *PTEN* loss may result in neuronal hypertrophy and other abnormalities^30,31,47^. This approach also avoids the risk of off-target mutagenesis and exogenous DNA integration that can be triggered by the Cas9 nuclease^48^. We designed a system using dCas9-KRAB to repress *PTEN* expression, and compared the extent of repression induced by the CRISPR system to that of four shRNAs targeting the *PTEN* transcript, which were previously shown to partially enhance optic nerve regeneration (Fig. 1C,D)^26^.

## Results

We investigated *PTEN* repression in human cell line models, neural stem cells and in induced pluripotent stem cell (iPSC)-derived CNS neurons using a CRISPR epigenetic editing system. We selected *S. pyogenes* dCas9 with a C-terminal KRAB fusion, which has been used previously for endogenous gene repression^44^. We designed four gRNAs targeting the *Homo sapiens PTEN* proximal promoter and 5′ untranslated region (UTR), two of which we had previously used for transcriptional activation of *PTEN*^49^ (Fig. 1C, Supplementary Table S1). gRNA target sites were selected for minimal predicted off-target activity and maximal on-target activity according to established algorithms^50^. We compared the extent of repression with dCas9-KRAB to that achieved from the delivery of four shRNAs targeting the *PTEN* transcript (Fig. 1D). Initially, the dCas9-KRAB system and *PTEN* shRNAs were tested in two model cell types—the human embryonic kidney (HEK) 293T cell line, and human mesenchymal precursor cells (hMPCs)—to establish the most effective gRNA before implementing the system in neural cells.

### dCas9-KRAB represses *PTEN* expression in the HEK 293T cell line and hMPCs

**Figure 2:**
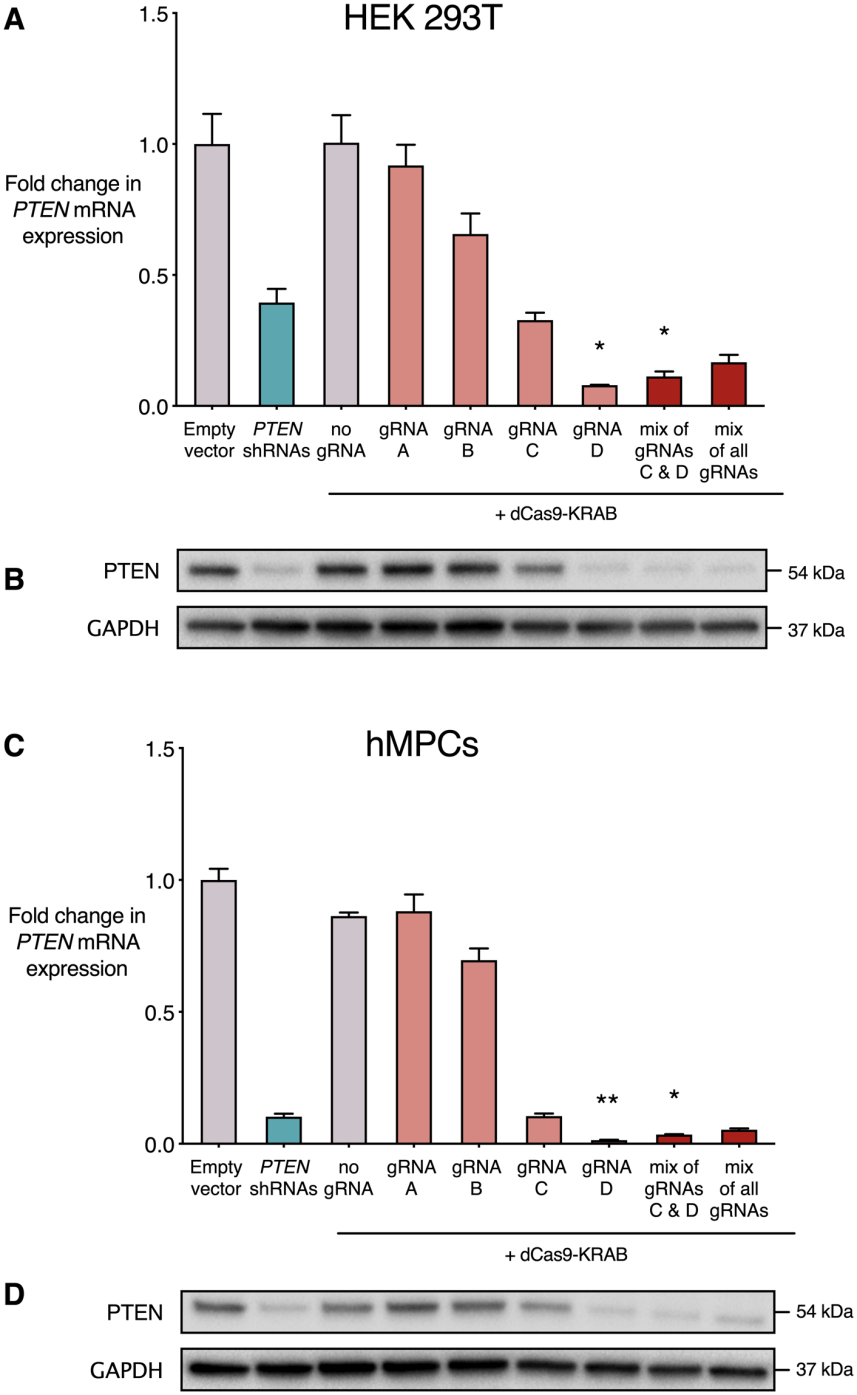
The dCas9-KRAB system represses *PTEN* in the HEK 293T cell line and human mesenchymal precursor cells (hMPCs). dCas9-KRAB was stably expressed with no gRNA, with individual gRNAs targeting the *PTEN* proximal promoter and 5′ untranslated region (UTR), with a mix of gRNAs C and D, or with a mix of all four gRNAs. Empty vector and the combination of four shRNAs targeting the *PTEN* transcript were also stably expressed by lentiviral transduction. (**A**,**C**) Fold change in *PTEN* mRNA expression in qRT-PCR relative to empty vector in the HEK 293T cell line (**A**) and hMPCs (**C**). *p < 0.05, **p < 0.01 (Kruskall–Wallis test with Dunn’s multiple comparisons test to compare each condition to empty vector control), n = 3, error bars show standard error of the mean (SEM). (**B**,**D**) Western blot of PTEN and GAPDH in HEK 293T (**B**) and hMPCs (**D**). Conditions correspond to qRT-PCR data labeled above.

The dCas9-KRAB system and *PTEN* shRNAs were delivered by lentiviral transduction and cell populations were collected and processed without selection for transduced cells. dCas9-KRAB was delivered with no gRNA or with individual *PTEN*-targeting gRNAs. We also tested the combined delivery of gRNAs C and D, which lie closest to the *PTEN* transcription start site (TSS), or a mix of all four gRNAs, as previous studies have shown more potent repression is sometimes achieved using multiple gRNAs per target gene^45,51–54^. qRT-PCR and Western blot were performed to assess *PTEN* mRNA and protein expression (Fig. 2A,B). In the HEK 293T cell line, *PTEN* expression was significantly repressed by gRNA D (0.08-fold, p < 0.05) and a combination of gRNAs C and D (0.11-fold, p < 0.05) relative to empty vector control. Interestingly, dCas9-KRAB repressed *PTEN* to a greater extent than the combination of four *PTEN* shRNAs (0.39-fold, p = 0.70). Delivering dCas9-KRAB with no gRNA, or any of the other individual gRNAs, did not result in a significant change in *PTEN* expression relative to the empty vector condition. The results of Western blot correlated with the strength of repression evident at mRNA level (Fig. 2B). A similar effect of these repression systems was observed in hMPCs. Relative to empty vector control, gRNA D (0.01-fold, p < 0.001) and the combination of gRNAs C and D (0.04-fold, p < 0.001) both showed significant *PTEN* repression (Fig. 2C). The relative level of *PTEN* expression in qRT-PCR results was reflected in Western blot (Fig. 2D).

### dCas9-KRAB does not induce transcriptional regulation at predicted off-target sites

**Figure 3:**
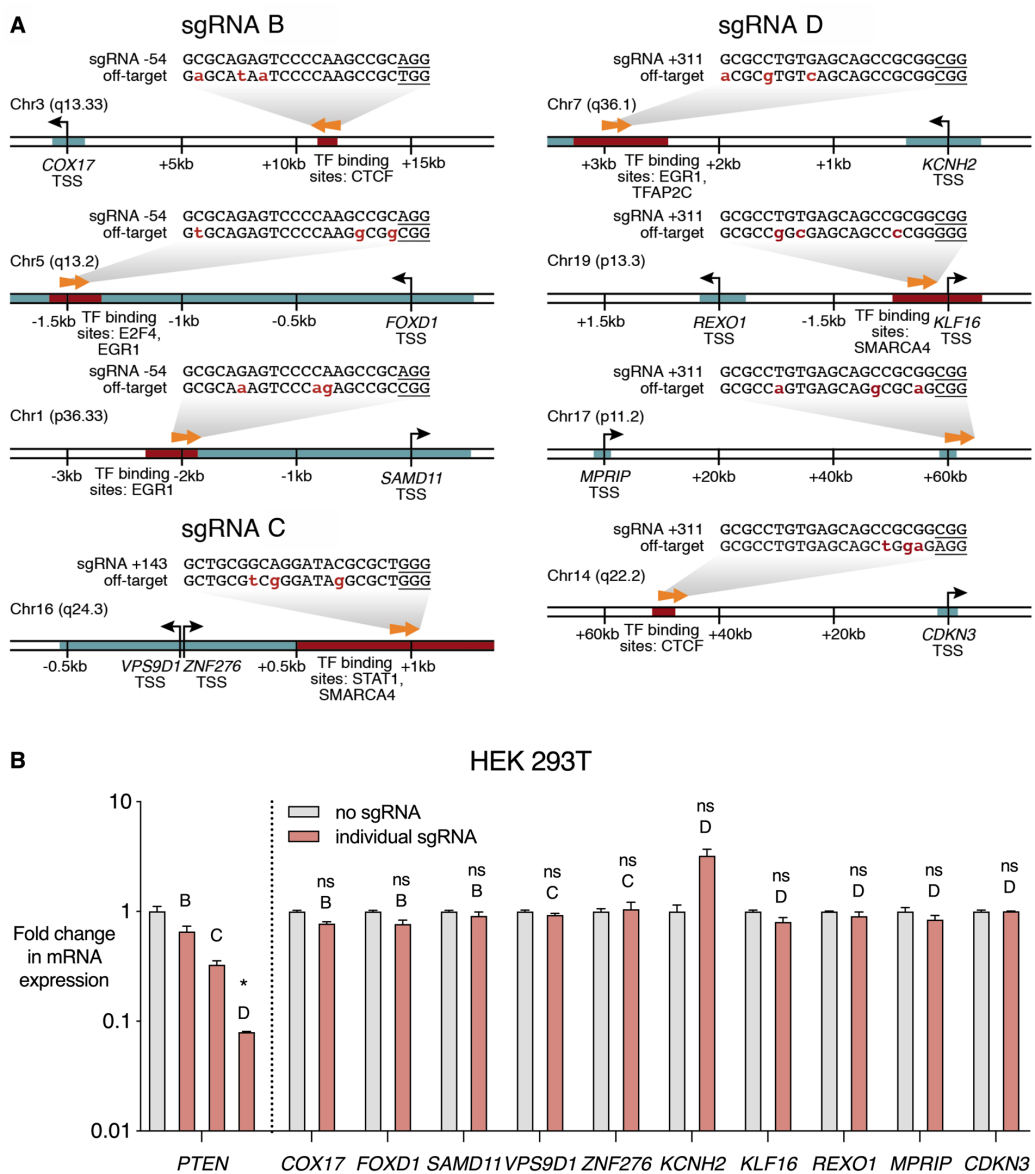
*PTEN* repression by dCas9-KRAB does not affect expression of predicted off-target genes. (**A**) Potential *S. pyogenes* Cas9 binding sites with 3 mismatches or less to the cognate sequence of any of the 4 *PTEN*-targeting gRNAs were identified as described previously^49^. Among all identified off-target sites, 8 were located in close proximity to regulatory elements and thus had increased potential to alter expression of the associated gene(s). The ten genes associated with these off-target sites were *COX17*, *FOXD1*, *SAMD11*, *VPS9D1*, *ZNF276*, *KCNH2*, *KLF16*, *REXO1*, *MPRIP* and *CDKN3*. PAM sequences are underlined and mismatches between cognate and off-target sequences are highlighted in red. Arrows indicate whether the gRNA targets the forward or reverse DNA strand. Numbering refers to the distance from the transcription start site (TSS) of the relevant gene. CpG islands are represented by green shaded regions of DNA, and transcription factor (TF) binding sites are represented by red shaded regions of DNA. (**B**) dCas9-KRAB was stably expressed in HEK 293T cell line with no gRNA or individual *PTEN*-targeting gRNAs. Data are shown as fold change in mRNA expression in qRT-PCR relative to dCas9-KRAB with no gRNA, for *PTEN* and each of the 10 potential off-target genes (PTEN expression data in Figure are reproduced from Fig. 2). The relevant PTEN-targeting gRNA (B, C or D) is indicated above the bar. No significant effect on mRNA expression was found for any predicted off-target gene. *p < 0.05 (Mann–Whitney test), n = 3, error bars show SEM.

Having established significant repression of *PTEN* with dCas9-KRAB, we then investigated whether the dCas9-KRAB system also induced off-target transcriptional regulation. We analysed *PTEN* gRNA sequences and compiled potential genome-wide off-target gRNA binding sites^55^. We then identified those off-target sites located in regulatory regions with the potential to modulate gene expression. Eight potential off-target binding sites were identified with proximity to regulatory elements of ten genes in total (Fig. 3A, Supplementary Table S2). qRT-PCR was conducted to assess regulation of these genes by dCas9-KRAB, comparing the relevant *PTEN*-targeting gRNA to dCas9-KRAB with no gRNA (Fig. 3B). There was no significant effect on expression of any of the potential off-target genes in HEK 293T cells transduced with *PTEN* repression components. Previous studies have also demonstrated negligible impact of CRISPR artificial transcription factors and epigenetic editors on gene expression or epigenetic modifications at off-target sites^45,53,56–66^.

### The dCas9-KRAB system elicits histone methylation and deacetylation at the *PTEN* transcriptional regulatory region

**Figure 4:**
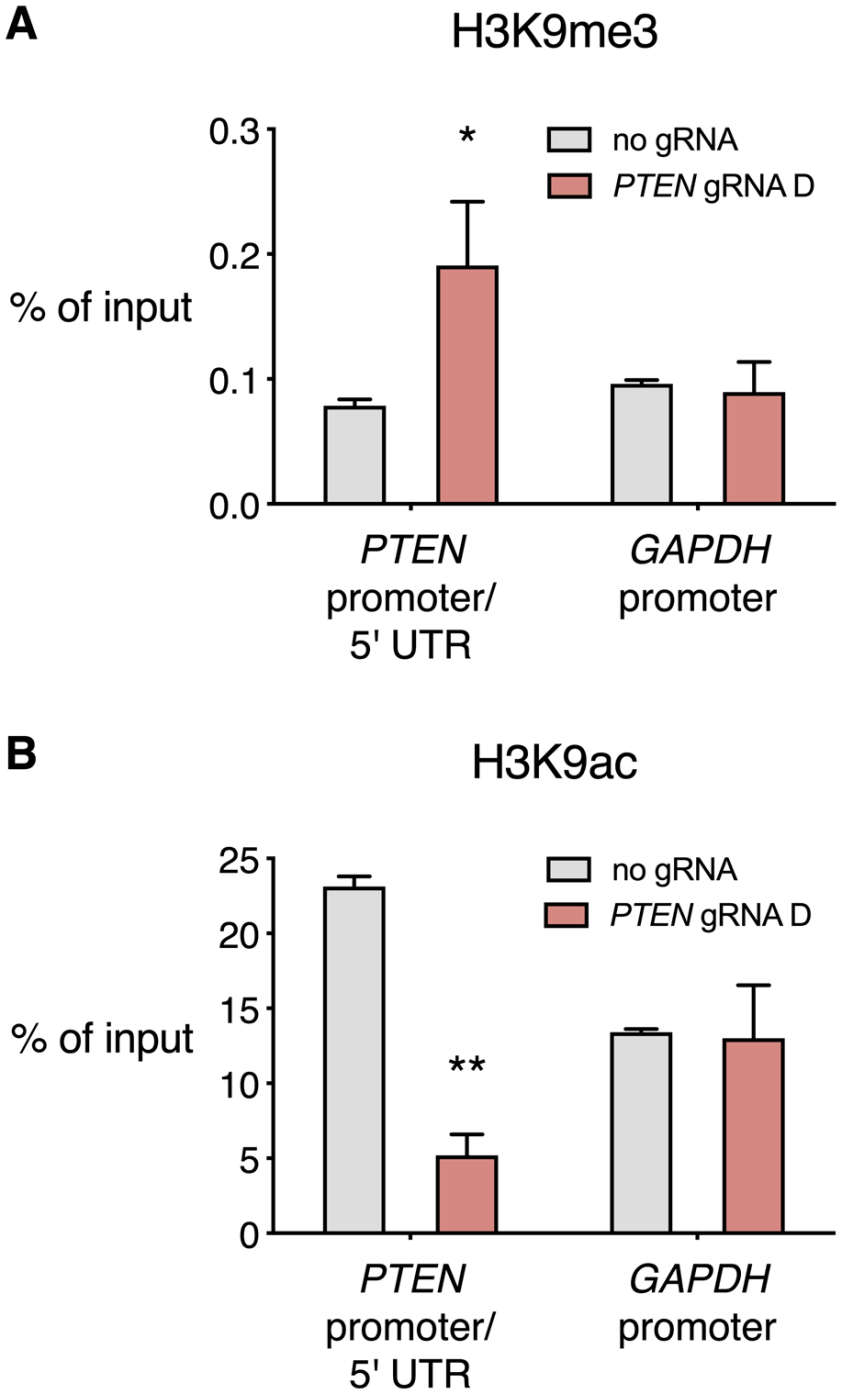
dCas9-KRAB alters histone modifications at the *PTEN* transcriptional regulatory region. (**A**,**B**) dCas9-KRAB was stably expressed in the HEK 293T cell line with gRNA D targeting the *PTEN* 5′ UTR, or with no gRNA. ChIP with H3K9me3 (**A**) and H3K9ac (**B**) antibodies was performed, followed by qPCR with primers in *PTEN* and *GAPDH* regulatory regions. Results are expressed as the percentage of immunoprecipitated DNA relative to input chromatin, *p < 0.05, **p < 0.01 (Student’s *t* test), n = 3, error bars show SEM.

dCas9-KRAB has previously been shown to recruit HDACs and HMTs resulting in changes to histone post-translational modifications at the target region^42,43^. We performed chromatin immunoprecipitation (ChIP) against trimethylated H3K9 (H3K9me3), a histone modification commonly associated with heterochromatin and transcriptional repression, and acetylated H3K9 (H3K9ac), which is highly correlated with active promoters (Fig. 4). We assessed H3K9me3 at a region spanning the junction of the *PTEN* proximal promoter and 5′ UTR, 70 base pairs upstream of the target site of *PTEN* gRNA D. H3K9me3 was significantly enriched in HEK 293T cells stably expressing dCas9-KRAB and *PTEN* gRNA D (0.19% of input chromatin compared to 0.08% of input chromatin with dCas9-KRAB with no gRNA, p < 0.05). H3K9ac was also significantly decreased at the *PTEN* transcriptional regulatory region with the expression of gRNA D (5.20% of input chromatin compared to 23.13% of input chromatin with dCas9-KRAB with no gRNA, p < 0.01). There were no significant differences in the frequency of H3K9me3 and H3K9ac at the *GAPDH* promoter between gRNA D and the no gRNA control condition, suggesting the epigenetic modifications induced by dCas9-KRAB and gRNA D were target gene specific.

### The dCas9-KRAB system induces *PTEN* repression in human iPSC-derived neural cells

**Figure 5:**
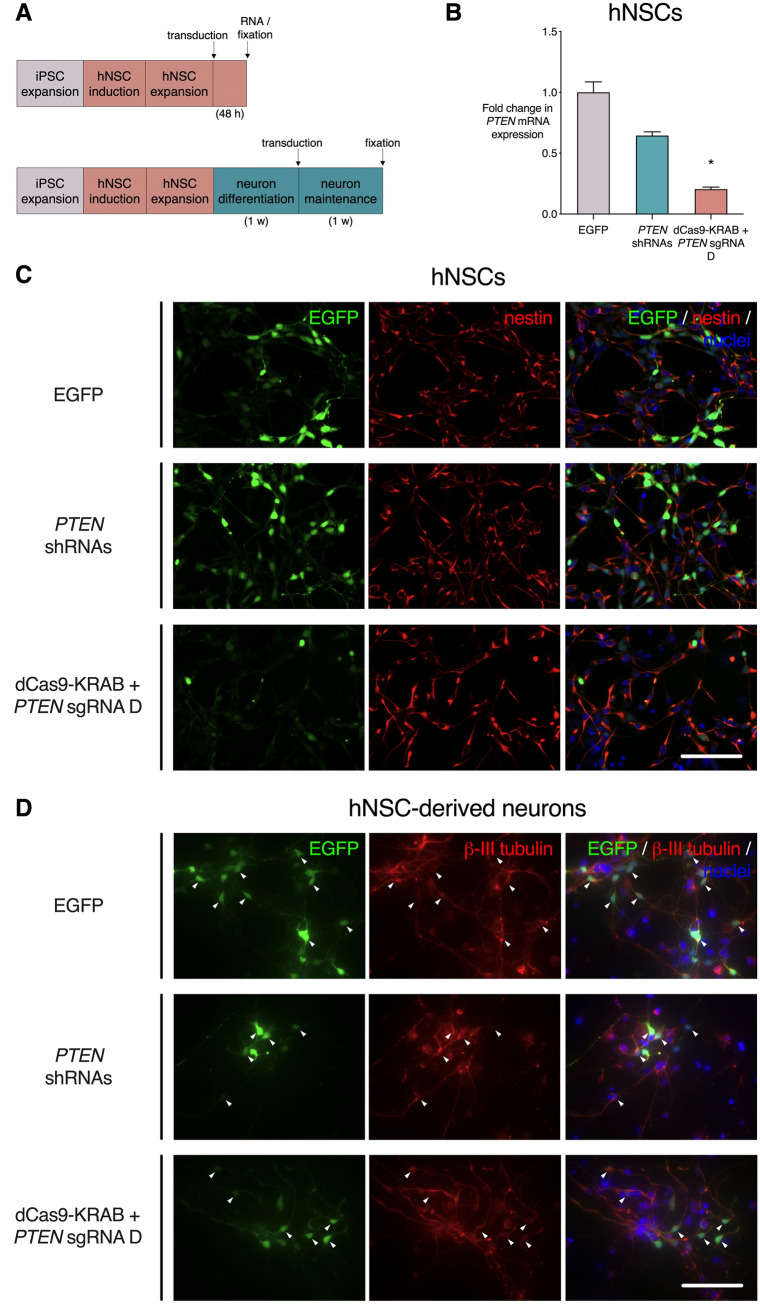
dCas9-KRAB represses *PTEN* in human iPSC-derived neural cells without altering cell identity. Human neural stem cells (hNSCs) or hNSC-derived neurons were transduced with lentivirus containing dCas9-KRAB and *PTEN* gRNA D, four shRNAs targeting *PTEN*, or EGFP control. (**A**) Schematic of iPSC to hNSC and neuron differentiation protocol. iPSCs were first expanded, followed by neural induction to generate hNSCs. Lentivirus was used to transduce hNSCs, followed by RNA extraction and fixation 48 h later (**A**, top). hNSCs were also differentiated into neurons for 1 week, followed by fixation 1 week later (**A**, bottom). (**B**) *PTEN* mRNA expression in unsorted hNSC populations 48 h post-transduction, shown as fold change relative to EGFP control. *p < 0.05 (Kruskall–Wallis test with Dunn’s multiple comparisons test to compare each condition to EGFP control), n = 3, error bars show SEM. (**C**,**D**) Representative images of transduced hNSCs (**C**) and neurons (**D**). Cells are stained for GFP and nuclear Hoechst 33,342, along with hNSC marker Nestin (**A**) and neuronal marker ßIII-tubulin (**C**). Scale bar = 200 µm.

Having identified the most potent gRNA for *PTEN* repression, we next delivered the lentiviral system in a neural cell model. Human neural stem cells (hNSCs) were derived from iPSCs originating from healthy donors, and were then transduced with equivalent titre of lentiviral particles expressing *PTEN* shRNAs, dCas9-KRAB and *PTEN* gRNA D, or EGFP control (Fig. 5A). RNA was collected from bulk cell populations without selection for transduced cells, and qRT-PCR was performed to assess levels of *PTEN* mRNA expression (Fig. 5B). In hNSCs, the delivery of four *PTEN* shRNAs reduced *PTEN* mRNA levels relative to empty vector control, but not significantly (0.65-fold, p = 0.34). However, dCas9-KRAB with *PTEN* gRNA D reduced *PTEN* expression significantly relative to empty vector control (0.20-fold, p < 0.05). Immunofluorescence analysis showed that transduction did not alter expression of the NSC marker Nestin in NSC populations (Fig. 5C). hNSCs were also differentiated to a neuronal phenotype over a period of 1 week, before delivering lentivirus encoding the shRNAs, the dCas9-KRAB system or EGFP control (Fig. 5A). Staining for the neuron-specific marker ßIII-tubulin showed that the majority of transduced (EGFP^+^) cells maintained a neuronal phenotype across all conditions (Fig. 5D).

### The dCas9-KRAB system induces *PTEN* repression in rat PC-12 cells

**Figure 6:**
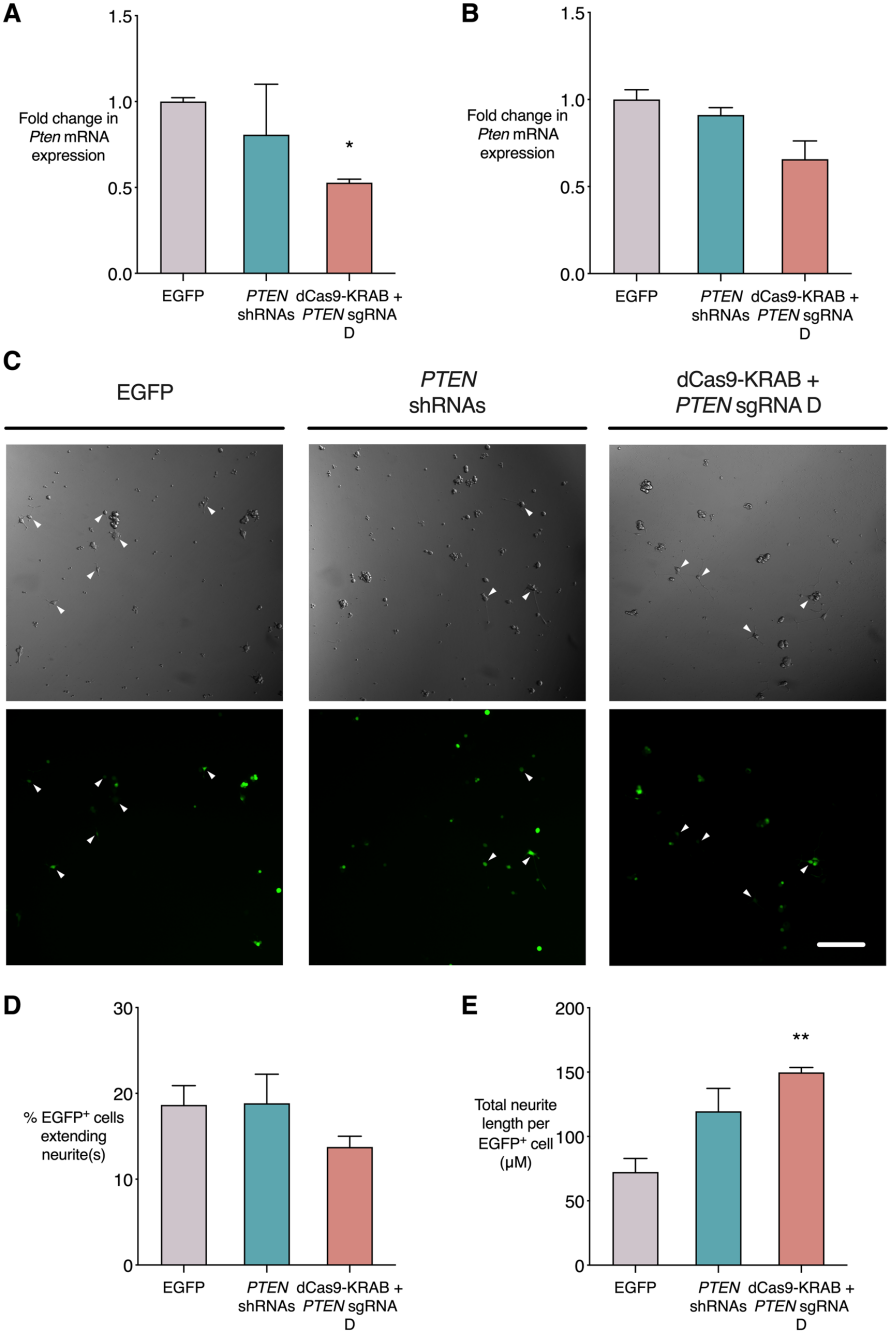
dCas9-KRAB represses *PTEN* and enhances neurite outgrowth in rat PC-12 cell line differentiated to neural phenotype. *Rattus norvegicus* PC-12 cells were transduced with dCas9-KRAB and *PTEN* gRNA D, four shRNAs targeting *PTEN*, or EGFP control, either before (**A**) or after (**B**–**E**) being differentiated to a neural phenotype with nerve growth factor (NGF). (**A**,**B**) *Pten* mRNA expression in qRT-PCR when virus was delivered to undifferentiated PC-12 cells (**A**) or to PC-12 cells that had already been stimulated with NGF for 48 h (**B**), shown as fold change relative to EGFP control, *p < 0.05 (Kruskall-Wallis test with Dunn’s multiple comparisons test to compare each condition to EGFP control), n = 3. (**C**) Representative images of differentiated PC-12 cells expressing EGFP, with phase contrast illustrating neurite length. Scale bar = 500 µm. (**D**) The percentage of transduced cells extending 1 or more neurites equal to or greater than the diameter of the cell body. (**E**) Total neurite length per differentiating, transduced cell. **p < 0.01 (One-way ANOVA with Dunnett’s multiple comparisons test to compare each condition to EGFP control), n = 3, error bars show SEM.

As the CRISPR/dCas9 system would ideally be tested in a preclinical rodent model of CNS injury, we wished to establish whether it could also successfully repress *Pten* in rat cells. gRNA D targets the *H. sapiens PTEN* 5′ UTR, and the binding site is perfectly conserved between human, rat and mouse, with no significant off-target sites present in the rat genome. In the rat, gRNA D binds to a site 679 base pairs upstream of the ATG initiation codon. We tested *Pten* repression in vitro in the rat PC-12 cell line, which is derived from adrenal medulla cells of neural crest origin. PC-12 cells stimulated with nerve growth factor (NGF) differentiate to a neural phenotype and extend neurites. Undifferentiated PC-12 cells (Fig. 6A) or PC-12 cells that had already been seeded and differentiated with NGF for 48 h (Fig. 6B) were transduced with *PTEN* shRNAs, dCas9-KRAB and *PTEN* gRNA D, or EGFP control. RNA from unselected populations of cells was extracted and qRT-PCR was performed to assess levels of *Pten* mRNA expression (Fig. 6A,B). In undifferentiated PC-12 cells, dCas9-KRAB with *PTEN* gRNA D significantly reduced *Pten* expression (0.53-fold, p < 0.05) relative to empty vector, while *PTEN* shRNAs did not significantly affect *Pten* mRNA levels (0.81-fold) (Fig. 6A). In PC-12 cells that had been stimulated with NGF for 48 h prior to transduction, the CRISPR repression condition showed lower *Pten* mRNA expression than EGFP control (0.66-fold), however this was not significant (p = 0.05) (Fig. 6B).

We also assessed the effect of *Pten* repression on the extent of neurite outgrowth in NGF-stimulated PC-12 cells. We transduced the PC-12 cell line 6 h after commencing induction of neurite outgrowth, then continued to stimulate with NGF until the extent of neurite outgrowth was assessed 4 days later (Fig. 6C–E). The percentage of transduced cells that extended neurites did not significantly differ between conditions (Fig. 6D). However, the average neurite length per differentiating cell was significantly increased with delivery of dCas9-KRAB and *PTEN* gRNA D, compared to EGFP control (149.8 µm compared to 72.3 µm in control, p < 0.01) (Fig. 6E). The superior performance of dCas9-KRAB repression to *Pten* shRNAs in rat neural-like cells is an encouraging finding when progressing to studies that will apply this method in preclinical CNS injury models.

## Discussion

We have shown that dCas9-KRAB targeted to the *PTEN* 5′ UTR by a single gRNA potently and specifically repressed *PTEN* expression at mRNA and protein level, and resulted in increased H3K9me3 and reduced H3K9ac at the *PTEN* transcriptional regulatory region. The dCas9-KRAB system repressed *PTEN* to a greater extent than a combination of four *PTEN*-targeting shRNAs in several experimental human cell types. Delivery of the CRISPR system to hNSCs or hNSC-derived neurons successfully repressed *PTEN* expression without altering expression of NSC-specific or neuron-specific markers. Previous studies have suggested that although *PTEN* shRNAs can partially improve the survival and axon regrowth of compromised neurons in vivo^23,25^, they are not as effective as *PTEN* genetic deletion, likely due to incomplete *PTEN* knockdown. The CRISPR/dCas9 system here achieved extremely potent repression in human cells, and could provide a strategy for *PTEN* inhibition that is almost as effective as *PTEN* genetic deletion, but with far greater translational potential, due to its reversibility and the reduced risk of exogenous DNA integration into Cas9-induced double-strand breaks^48^. In addition, there are some concerns as to significant levels of off-target activity produced by RNA interference strategies. The gRNAs employed here did not affect expression of predicted off-target genes, and many other studies support the claim that transcriptional regulation with dCas9 is highly specific^45,53,56–65^.

Cultured neurons do not provide definitive information as to whether dCas9-KRAB can promote axon regeneration of damaged CNS neurons in vivo, thus the CRISPR system must eventually be tested in a preclinical, most likely rodent, model of CNS injury. The gRNA sequence we used is conserved in rat and mouse, and the same CRISPR system that worked effectively in human cells also significantly downregulated *Pten* and increased neurite outgrowth in rat PC-12 neural crest-derived cells. The efficacy of CRISPR *Pten* repression in PC-12 cells suggests that the system might be effectively applied in rat CNS injury models in the future. We packaged dCas9-KRAB and gRNA in lentivirus; however recombinant AAV (rAAV) vectors are generally thought to be a safer alternative for clinical gene therapy applications as they present lower risk of insertional mutagenesis from viral DNA integration^67^. rAAV DNA molecules persist predominantly as episomes in vivo, with relatively rare instances of integration into the host genome, and have been approved for clinical trials^68^. In addition to the size of the therapeutic gene, the packaging limit of AAV complicates the accommodation of *S. pyogenes* dCas9, which is 4.1 kilobase pairs prior to the addition of promoters, effector domain, and gRNA required for transcriptional repression. To address this, dual AAV systems have been used for CRISPR gene editing and transcriptional regulation in mice and primates^69–73^. In the context of nervous system repair, a promising approach for *PTEN* repression would be to deliver dCas9-KRAB in single-stranded AAV, along with CRISPR gRNA in self-complementary AAV, as has been demonstrated recently^74^. In addition, Cas proteins from other species, such as *Staphylococcus aureus* dCas9, are small enough to be accommodated in AAV for transcriptional activation and repression, and these could prove to be an effective alternative to *S. pyogenes* dCas9 for in vivo delivery^75,76^.

In both preliminary cell types tested, HEK 293T and hMPCs, combining multiple *PTEN*-targeting gRNAs did not achieve greater levels of repression than the single most potent gRNA. This single gRNA also achieved significant *PTEN* repression in hNSCs and PC-12 cells. This is an encouraging finding as it is easier to deliver a single gRNA when moving to in vivo applications, and also promotes the possibility of multiplex transcriptional regulation by delivering several gRNAs, each targeting a different gene^77–80^. gRNA multiplexing is of special interest considering *PTEN* deletion has previously been shown to cooperate with deletion of other growth-suppressing genes to achieve even greater levels of axon regeneration in the CNS. For example, co-deletion of *PTEN* and suppressor of cytokine signaling 3 (*SOCS3)*, a key negative regulator of the signal transducer and activator of transcription (STAT) pathway, improved axon regeneration to an even greater extent than *PTEN* deletion alone^18,81^.

Bisperoxovanadium (bpV) compounds, inhibitors of protein tyrosine phosphatases (PTPs), have also been used for pharmacological modulation of PTEN in the context of spinal cord injury and ischemia^82–84^. Although bpV compounds exhibit some level of selectivity for PTEN, they also block other PTPs at higher concentrations, and so may present a risk of unintended non-specific effects. Systemic administration of bpV PTEN inhibitors may also have unintended effects in non-neuronal cell types, whereas AAV tropism and cell type-specific promoters provide a means to limit expression of the CRISPR system to neurons^85^.

Although a potentially powerful tool in CNS repair strategies, *PTEN* repression may not be safe or desirable beyond the point of axon regeneration and the reformation of connections with target neurons. There is the possibility that constitutive and permanent *PTEN* knockdown could lead to cancer development, as *PTEN* has well-established tumor suppressive functions^49,86^. Importantly, there is evidence that conditional *PTEN* deletion in mature neurons causes progressive growth of axons and dendrites, and hypertrophy of cell bodies^30,31,47^. These considerations suggest that temporal regulation of *PTEN* repression would be a safer and more clinically relevant approach. Because the transcriptional repression induced by epigenetic CRISPR/dCas9-KRAB editing does not persist in absence of continued effector expression^64,87^, the methodology described in the present study represents a promising alternate strategy to silence *PTEN* without permanent gene knockout. In addition, epigenetic editing avoids the risk of off-target mutagenesis and exogenous DNA integration that is associated with gene editing^48^. One promising approach may be to obtain transient expression of CRISPR repression components, by using a system that can be chemically controlled either at the transcriptional^85,88–90^ or posttranslational level^90^.

It is important to note that the regrowth of axons is only the first step in restoring CNS function. Although axon regeneration after *PTEN* deletion results in functional improvements^23,25,84^, there is also evidence of targeting errors by regenerating *PTEN*-deleted axons^91,92^, another reason for temporal modulation of *PTEN* repression. Target innervation is a complex process delicately orchestrated by developmental guidance cues that are usually absent in the adult^93^. Establishing synaptic maps that provide functional recovery is another hurdle to overcome once axon extension is achieved, and may require the exogenous delivery of branching-promoting factors, as well as other neurotrophins and guidance cues^4,94,95^. Rehabilitative training may also be necessary to promote synaptogenesis following axon regeneration. In summary, while there are many obstacles still involved in overcoming regenerative failure in the adult CNS, application of CRISPR/dCas9 technology for *PTEN* repression may prove to be an effective, and regulatable, approach to combating its debilitating effects.

## Methods

### Cell culture

The human embryonic kidney (HEK) 293T cell line was obtained from the American Type Culture Collection (ATCC, Manassas, VA) and cultured in DMEM (produced by the Harry Perkins Institute of Medical Research, Perth, Australia; formulated to ATCC specifications) supplemented with 10% heat-inactivated HyClone Fetal Bovine Serum (FBS; Thermo Fisher Scientific, Waltham, MA) and 1% Antibiotic–Antimycotic (Gibco, Fisher Scientific, Hampton, NH). hMPCs were a gift from Dr. Marian Sturm at Cell & Tissue Therapies WA (CTTWA), Royal Perth Hospital. hMPCs were isolated by CTTWA from healthy donors at Royal Perth Hospital, Perth, Australia, using Ficoll-Paque density centrifugation and plastic adherence in culture, and expressed MPC surface markers. hMPCs were cultured in ATCC-formulated MEM alpha (Harry Perkins Institute of Medical Research) supplemented with 10% heat-inactivated HyClone FBS and 1% Antibiotic–Antimycotic. The PC-12 cell line was obtained from the ATCC and cultured in ATCC-formulated RPMI-1640 medium (Harry Perkins Institute of Medical Research) supplemented with 10% horse serum, 5% heat-inactivated HyClone FBS and 1% Antibiotic–Antimycotic.

The Human Episomal iPSC Line (Gibco) was expanded in Essential 8 Medium (Gibco) and differentiated to neural stem cells (hNSCs) and expanded according to the manufacturer’s protocol. After expansion, hNSCs were cultured on plates coated with Geltrex hESC-Qualified, Ready-To-Use, Reduced Growth Factor Basement Membrane Matrix (Gibco), in medium containing 2% Neural Induction Supplement (Gibco) and 1:1 mix of Neurobasal Medium (Gibco) and Advanced DMEM/F-12 (Gibco). For differentiation into neurons, hNSCs were seeded onto plates coated with 0.05% Poly(ethyleneimine) solution (Merck) and 3.3 μg/mL laminin, in the following medium: 2% B-27 Supplement, serum free (Gibco), 1% N-2 Supplement (Thermo Fisher), 1% GlutaMAX (Gibco), 1% Insulin-Transferrin-Selenium-Sodium Pyruvate (ITS-A) (Gibco), 10 ng/mL Recombinant Human β-NGF (Peprotech, London, United Kingdom), 10 ng/mL Recombinant Human NT-3 (Peprotech), 10 ng/mL Recombinant Human/Murine/Rat BDNF (Peprotech), in 1:1 mix of Neurobasal Medium (Gibco) and Advanced DMEM/F-12 (Gibco). After commencing differentiation, cells were maintained in differentiation medium for 1 week prior to lentiviral transduction.

### gRNA and shRNA design

#### gRNA target design and off-target identification

Candidate gRNA sequences for *PTEN* repression were identified using the Benchling CRISPR design tool (benchling.com), which provides a score indicating the predicted targeting specificity and off-target binding sites of each gRNA according to established algorithms^50,96^. gRNAs were only considered if they had a specificity score greater than 60 and an efficiency score greater than 40. Forty-four putative gRNAs were available in the region starting 400 bp upstream of the transcription start site (TSS), and extending 400 bp downstream into the 5′ UTR of *PTEN* mRNA transcript variant 1. From these, 4 gRNAs were selected which had specificity and efficiency scores above the designated thresholds, and which were relatively evenly spaced across the target region. The four gRNA target sequences chosen for *PTEN* repression, along with their specificity and efficiency scores, are listed in Supplementary Table S1.

To identify potential off-target gRNA binding sites with the potential to modulate gene expression, the software program Cas-OFFinder^55^ was used to search for genomic sequences that were highly similar to any of the 4 *PTEN* gRNAs, and upstream of the *S. pyogenes* NGG PAM. The search was restricted to off-target sites with three mismatches or less to the corresponding cognate gRNA sequence. The location of each potential off-target site was compared to UCSC Genome Browser and ENCODE data, to identify proximity to annotated NCBI RefSeq genes, promoters, enhancers, CpG islands, DNase I hypersensitive regions and transcription factor binding sites, which would indicate greater potential for the dCas9-KRAB complex to modulate gene expression. Eight off-target sequences were found to fall in close proximity to potential regulatory elements of ten genes in total (Supplementary Table S2), and were assessed by qRT-PCR as described below.

#### shRNA design

*PTEN* shRNAs were based on SIBR vectors in which shRNA is located in an intron and flanked by sequences derived from miRNA-155, an endogenous intronic shRNA. Four separate shRNA sequences, each targeting a different region of *PTEN*, were concatenated in a single plasmid^26^. The four shRNA sequences are listed in Supplementary Table S3. shRNA + 2022 contained one nucleotide mismatch to the *H. sapiens PTEN* transcript, as this shRNA vector was originally designed to target rat and mouse *Pten*. However, there were no significant off-target sequences for these shRNAs identified in the human transcriptome.

### Plasmids

For validating individual gRNAs for repression, each gRNA was cloned into the pLV hU6-sgRNA hUbC-dCas9-KRAB-T2A-Puro third-generation lentiviral transfer plasmid^44^ (Addgene plasmid #71236, a gift from Charles Gersbach; hereafter referred to as pLV-KRAB). Cloning of annealed gRNA oligonucleotides into BsmBI sites was carried out as described previously^97^. After establishing that gRNA D achieved optimal gene repression, the gRNA D target sequence was cloned into the pLV hU6-sgRNA hUbC-dCas9-KRAB-T2a-GFP third-generation lentiviral transfer plasmid^44^ (Addgene plasmid #71237, a gift from Charles Gersbach; hereafter referred to as pLV-KRAB-EGFP). pLV-KRAB and pLV-KRAB-EGFP encode humanized *S. pyogenes* dCas9 protein (with mutations in D10A and H840A) under the control of the hUbC promoter, along with the gRNA scaffold under the control of the hU6 promoter.

pLenti-shPTEN-EGFP was cloned to express four shRNAs targeting the *PTEN* transcript. The shRNAs were sourced from pAAV-shPTEN plasmid, a gift from Dr. Murray Blackmore (Marquette University) and Kevin Park. A region of pAAV-shPTEN comprising the ubiquitin promoter, intronic sequences, knockdown cassette, and EGFP open reading frame was cloned into the pLenti backbone (pLenti-dCAS-VP64_Blast, Addgene plasmid #61425) using NEBuilder HiFi DNA Assembly Master Mix (New England Biolabs, Ipswich, MA). pLenti-EGFP was cloned by removing the *PTEN* shRNA cassette from pLenti-shPTEN-EGFP.

pLenti-CMV_Blast_empty^98^ (Addgene plasmid #17486) and pLV-KRAB with no inserted gRNA sequence were used as controls for experiments in HEK 293T and hMPCs. The third-generation lentiviral packaging plasmids pMDLg/pRRE (Addgene plasmid #12251) and pRSV-Rev (Addgene plasmid #12253), and envelope plasmid pMD2.G (Addgene plasmid #12259; all gifts from Didier Trono), were used for lentiviral production.

### Lentiviral production and transduction

Experiments were approved by the Australian Office of the Gene Technology Regulator (OGTR) under Notifiable Low Risk Dealing (NLRD) approval number 004/2017. Lentivirus was produced by transfection of HEK 293T cells with lentiviral transfer, packaging and envelope plasmids as described previously^99^. Briefly, HEK 293T cells were seeded in 10 cm plates (4 × 10^6^ cells per plate) one day prior to transfection. Cells were transfected with packaging, envelope and transfer plasmids (described above) using Lipofectamine 3000 Transfection Reagent (Thermo Fisher) according to the manufacturer’s protocol, using 8.4 μg packaging plasmid, 5.5 μg envelope plasmid and 10.9 μg transfer plasmid DNA per plate. Cells were incubated in transfection mixture overnight and the medium was changed the following morning. Supernatant containing lentiviral particles was removed at 48 and 72 h post-transfection, and supernatant from the two collection times was pooled before concentration.

Supernatant containing viral particles was concentrated by adding 1 volume of 40% Polyethylene glycol 8,000 (Sigma-Aldrich, St. Louis, MO), 1.2 M NaCl, pH 7.0 solution to 3 volumes of supernatant, shaking at 60 rpm overnight at 4 °C, followed by centrifugation at 1,600 RCF for 1 h at 4 °C. The supernatant was then removed and the pellet containing lentiviral particles was resuspended in fresh culture medium specific to the cell type of interest. Lentivirus was titrated based on EGFP expression as assessed by flow cytometry. For transduction, lentivirus was added to the culture medium overnight along with Polybrene Infection/Transfection Reagent (Merck Millipore, Burlington, MA; 5 μg/mL for hNSCs and neurons, 8 μg/mL for all other cell types) and exchanged for fresh culture medium the following morning.

### RNA extraction, reverse transcription and qRT-PCR

RNA was extracted from transduced cells using phenol–chloroform extraction with QIAzol Lysis Reagent (QIAGEN, Hilden, Germany)^49^. Purified total RNA (2 μg for HEK 293T, hMPCs and PC-12, and 250 ng for hNSCs) was used to generate cDNA using the High Capacity cDNA Reverse Transcription Kit (Applied Biosystems, Foster City, CA), with incubation at 25 °C for 10 min, followed by 37 °C for 120 min, and inactivation at 85 °C for 5 min.

Real-time quantitative reverse transcription PCR (qRT-PCR) was performed as described previously^49^. qRT-PCR for genes *PTEN*, *GAPDH*, *KLF16* and *SAMD11* was conducted with TaqMan Gene Expression Assays (Applied Biosystems), listed in Supplementary Table S4. qRT-PCR for genes *COX17*, *FOXD1, SAMD11*, *VPS9D1*, *ZNF276*, *KCNH2*, *MPRIP*, *CDKN3* and *Pten* (rat) was conducted with QuantiFast SYBR Green PCR Master Mix (QIAGEN) and custom designed primers (Integrated DNA Technologies, Coralville, IA), listed in Supplementary Table S5. The ViiA 7 Real-Time PCR System (Applied Biosystems) was used to carry out the qRT-PCR reactions. Thermocycling settings for TaqMan assays were: 95 °C for 20 s, followed by 40 cycles of 95 °C for 1 s and 60 °C for 20 s. Thermocycling settings for SYBR Green assays were: 95 °C for 5 min, followed by 40 cycles of 95 °C for 10 s and 60 °C for 30 s. This was followed by a melt curve program: 95 °C for 15 s, 60 °C for 1 min, and a ramp of 0.05 °C per second to 95 °C. QuantStudio Real Time PCR Software (v1.1, Applied Biosystems) was used to automatically determine cycle threshold (Ct) for each well. Relative quantification of gene expression was analyzed using the comparative (ΔΔ) Ct method^100,101^ with *GAPDH* or *Ppia* as housekeeping gene.

### Protein extraction and quantification

Protein extraction from transduced cells was performed using Cell Lysis Buffer (Cell Signaling Technology, Danvers, MA)^49^. Samples were sonicated for 15 s at 10 mA, followed by centrifugation at 16,000 RCF for 10 min at 4 °C, and transferal of supernatant to a new tube. Samples were quantified with the DC Protein Assay (Bio-Rad, Hercules, CA) using the recommended protocol. Sample absorbance at 750 nm was quantified using the PowerWave XS2 Microplate Spectrophotometer (BioTek, Winooski, VT).

### Western blotting

Western blotting was carried out as described previously^49^. Proteins were resolved with Mini-PROTEAN TGX Stain-Free Protein Gels (Bio-Rad), loading 20 μg of protein per lane. The TransBlot Turbo (Bio-Rad) was used to transfer proteins to a 0.2 μM PVDF membrane (Trans-Blot Turbo Transfer Pack, Bio-Rad). Membranes were blocked using 5% skim milk powder in tris-buffered saline with Tween-20 (Sigma-Aldrich) (TBS-T) for 1 h at room temperature with gentle shaking. Following blocking, membranes were incubated with primary antibody in TBS-T at 4 °C overnight (antibodies are listed in Supplementary Table S6). The following day, membranes were washed and incubated with secondary antibody in TBS-T for 1 h at room temperature. Blots were visualized with Luminata Crescendo Western HRP Substrate (Merck-Millipore) using the ChemiDoc MP system (Bio-Rad) and ImageLab Software (Bio-Rad). Images of uncropped Western blots from Fig. 2 are displayed in Supplementary Fig. S1.

### Chromatin immunoprecipitation (ChIP)-qPCR

ChIP was carried out as described previously^102^. Briefly, samples were cross-linked in 1% formaldehyde for 10 min at room temperature with gentle rocking. Cross-linking was quenched by adding 100 μL of 1.375 M glycine per milliliter of culture. Samples were washed and collected in ice-cold PBS, followed by cell lysis and collection of nuclei according to the Cold Spring Harbor (CSH) ChIP protocol^102^. Nuclear pellets were sonicated in the Covaris M220 Focused-ultrasonicator (Thermo Fisher Scientific) in 1 mL Covaris milliTUBEs (Thermo Fisher Scientific) at 75 W peak incident power, 10% duty factor and 200 cycles per burst for 9 min at 7 °C. Pulldown was conducted according to the CSH protocol^102^ using Dynabeads Protein G (Invitrogen, Carlsbad, CA) and Tri-Methyl-Histone H3 (Lys9) and Acetyl-Histone H3 (Lys9) rabbit monoclonal antibodies (Cell Signaling Technology, #13969 and #9649), with no antibody as control. 1% of input chromatin was reserved as input control. DNA was purified from immunoprecipitated samples by phenol–chloroform-isoamyl alcohol DNA extraction.

Real-time quantitative PCR (qPCR) was performed on purified DNA samples with primers for the *PTEN* regulatory region and *GAPDH* promoter, and SYBR Green Quantifast PCR Master Mix. *PTEN* primers were as described previously^103^. Primers are listed in Supplementary Table S7. The reaction was carried out in the ViiA 7 Real-Time PCR System (Applied Biosystems) with the following thermocycling settings: 95 °C for 5 min, followed by 40 cycles of 95 °C for 10 s and 60 °C for 30 s. This was followed by a melt curve program: 95 °C for 15 s, 60 °C for 1 min, and a ramp of 0.05 °C per second to 95 °C. Cycle threshold (Ct) was automatically determined for each well using QuantStudio Real Time PCR Software (v1.1, Applied Biosystems). Quantification was performed according to the percent input method, in which signals obtained from the ChIP are divided by signals obtained from the input sample. Percent input values for each condition were calculated according to the formula:

$$100 {\times 2}^{(Adjusted Input Ct-IP Ct)}$$

where Adjusted Input Ct is the Ct obtained from the 1% input chromatin sample, adjusted for dilution factor, and IP Ct is the Ct obtained from the IP for that condition. This method corrects for variations in the amount of chromatin used in ChIP for each condition.

### Immunofluorescence

Cells plated on Geltrex or PEI-laminin coated plates were fixed with 4% formaldehyde in DPBS for 20 min at room temperature. For immunostaining, samples were blocked with 5% normal goat serum (Invitrogen) and 0.3% Triton X-100 (Sigma-Aldrich) in DPBS for 1 h at room temperature. Samples were then incubated with primary antibodies (GFP: Roche #11814460001; nestin: Biolegend #841901; ßIII-tubulin: Biolegend #802001) in diluent buffer (1% BSA and 0.3% Triton X-100 in DPBS) at 4 °C overnight. Full details of antibodies and dilution factors used for immunofluorescence are listed in Supplementary Table S8. Following overnight incubation, samples were washed and incubated with secondary antibody in diluent buffer for 2 h at room temperature, protected from light. Slides and coverslips were mounted using SlowFade Diamond Antifade Mountant (Molecular Probes, Eugene, OR). Images were acquired with the Olympus DP71 fluorescent microscope and DP Controller and DP Manager software (Olympus, Shinjuku, Japan).

### Neurite outgrowth

For neurite outgrowth assays, PC-12 cells were seeded in PC-12 differentiation medium at a density of 2.6 × 10^4^ cells per well in 24-well plates, coated with Poly-l-lysine (Sigma-Aldrich). PC-12 differentiation medium consisted of 1% horse serum and 1 ng/mL β-NGF (Peprotech) in RPMI-1640 medium (Harry Perkins Institute of Medical Research). Six hours after seeding, cells were transduced with lentivirus as described above. Cells were incubated with lentivirus overnight and the following day, transduction medium was replaced with PC-12 differentiation medium. Images were acquired four days after cells were initially seeded. Four fields of view were analyzed from each well, with 3 wells analyzed per condition. Images were quantified using ImageJ software (NIH).

### Statistical analysis

Statistical analyses were performed with Prism 8 (GraphPad Software Incorporated, La Jolla, CA). Statistical significance of qRT-PCR data was determined by non-parametric Kruskall–Wallis and Mann–Whitney tests. Statistical significance for all other data was determined using one-way ANOVA or Student’s *t*-tests. For Kruskall–Wallis tests and one-way ANOVA, post-hoc multiple comparisons tests were performed to compare the mean of each experimental condition to the control condition. Differences were considered significant at p < 0.05 (*) and p < 0.01 (**). Error bars show standard error of the mean (SEM).

## Supplementary information

Supplementary Information

## Data Availability

Plasmids generated for the current study are available from the corresponding authors on reasonable request. All data generated or analysed during this study are included in this published article (and its Supplementary Information files).
