# Supplementary material for: Transcriptional repression of PTEN in neural cells using CRISPR/dCas9 epigenetic editing
Source: Sci Rep. 2020 Jul 9;10:11393. doi: 10.1038/s41598-020-68257-y (PMC7347541; doi:10.1038/s41598-020-68257-y)
Supplement: Supplementary file 1 — Supplementary Information [file 41598_2020_68257_MOESM1_ESM.pdf]

Supplementary information

Transcriptional repression of *PTEN* in neural cells using CRISPR/dCas9 epigenetic editing

Moses, C., Hodgetts, S. I., Nugent, F., Ben-Ary, G., Park, K. K., Blancafort, P.*, Harvey, A. R.*

* To whom correspondence should be addressed. E-mail: alan.harvey@uwa.edu.au; pilar.blancafort@uwa.edu.au

Supplementary Table 1. gRNA recognition sequences for *PTEN* repression. The numbering of the start and end of each gRNA is given according to the Genome Reference Consortium Human Build 38 (GRCh38). PAM: protospacer-adjacent motif; F: forward strand guide; R: reverse strand guide. Location refers to the genomic position of the gRNA binding site in base pairs upstream (-) or downstream (+) the transcription start site of *PTEN* transcript NM_000314.8 in GRCh38.

gRNA	crRNA recognition sequence	PAM	F/R	Start	End	Location
A	GAGGATAACGAGCTAAGCCT	CGG	R	87863256	87863237	-368
B	GCGCAGAGTCCCCAAGCCGC	AGG	R	87863383	87863364	-241
C	GCTGCGGCAGGATACGCGCT	CGG	F	87863580	87863599	-45
D	GCGCCTGTGAGCAGCCGCGG	GGG	F	87863748	87863767	+123

Supplementary Table 2. Analysis of predicted off-target binding sites of *PTEN* gRNAs. In the off-target recognition sequence, mismatches to the cognate gRNA sequence are shown in lower case and underlined. Position refers to chromosomal location in GRCh38. Distances from the TSS of off-target genes are approximate and refer to annotated RefSeq transcripts. Literature-curated TF binding sites and associated genes are from the PAZAR and JASPAR databases. Regions of H3K4me3, H3K4me1 and H3K27ac enrichment are obtained from the ENCODE project. Chr: chromosome; TF: transcription factor; TSS: transcription start site.

gRNA	Off-target recognition sequence	Chr	Position	Direction	Mismatches	Proximity to regulatory elements	Potential gene affected
A	GAGGATAACGAGCccAGCCaGGG	19	16105684	+	3	None	None
B	GaGCAtAaTCCCCAAGCCGCTGG	3	119690285	-	3	12 kb upstream of *COX17* TSS; overlaps CTCF TF binding sites for *COX17*; in DNase I hypersensitive region	*COX17*
	GCtCAGAGTCCCCAAGgCcCTGG	3	140566600	+	3	None	None
	GCGCtGAGTCCCCAAGCaGaTGG	4	8738833	+	3	None	None
	GtGCAGAGTCCCCAAGgCGgCGG	5	73447021	+	3	1.5 kb downstream of *FOXD1* TSS; in CpG island; overlaps E2F4 and EGR1 TF binding sites for *FOXD1*; in DNase I hypersensitive region; in region of H3K27ac, H3K4me3, H3K4me1 enrichment	*FOXD1*
	GCGCAaAGTCCCagAGCCGCCGG	1	923628	+	3	2 kb upstream of *SAMD11* TSS; in CpG island; overlaps EGR1 TF binding sites for *SAMD11*; in region of H3K4me3, H3K4me1 enrichment	*SAMD11*
	GCtCAGAGaCCCCAAGCCtCGGG	1	3983348	-	3	None	None
	GtGCtGAGTCCCCAAGCCcCAGG	9	136764513	+	3	None	None
C	GCTGCGtCgGGATAgGCGCTGGG	16	89721972	+	3	1 kb upstream of *VPS9D1* TSS; overlaps SMARCA4 TF binding	*VPS9D1*; *ZNF276*

						site for VPS9D1; 1 kb downstream of *ZNF276* TSS; overlaps STAT1 TF binding site for *ZNF276*; in region of H3K27ac, H3K4me3, H3K4me1 enrichment	
	GCTGaGGCAGGAgACtCGCTTGG	1	1497763	+	3	None	None
	GCTGCaGCAGGATACaCcCTGGG	1	18823163	+	3	None	None
	GCTGgGGCAGGATAatCGCTTGG	13	34275296	+	3	None	None
	GCTGCGGCAGGAgAatCGCTTGG	13	110571118	+	3	None	None
	GCTGaGGCAGGAgACtCGCTTGG	10	86424569	+	3	None	None
	GCTGaGGCAGGAaACtCGCTTGG	10	110432686	-	3	None	None
	GCTGaGGCAGGATAatCGCTCGG	18	50856030	-	3	None	None
	GCTGCaGCAtGATACGtGCTAGG	11	50193345	+	3	None	None
D	aCGCgTGTcAGCAGCCGCGGCGG	7	150975260	+	3	3 kb downstream of *KCNH2* TSS; overlaps EGR1 and TFAP2C TF binding sites for *KCNH2*; in region of H3K27ac, H3K4me3, H3K4me1 enrichment	*KCNH2*
	GCcCCTGTGAGCAGCCtCtGTGG	4	8025678	-	3	None	None
	GCcCCTGTGAGCAGCtGCaGGGG	16	897591	+	3	None	None
	GCcCCTGTGgGCAGCCaCGGGGG	1	2177765	-	3	None	None
	GCGCCgGcGAGCAGCCcCGGGGG	19	1851994	+	3	0.4 kb upstream of *KLF16* TSS; in CpG island; overlaps SMARCA4 TF binding site for *REXO1*; in DNase I hypersensitive region	*KLF16*; *REXO1*

	GCGCCaGTGAGCAGgCGCaGCGG	17	17164604	+	3	60 kb downstream of *MPRIP* TSS; in CpG island	*MPRIP*
	GCtCCTGTcAGCAGCtGCGGAGG	10	129709862	-	3	None	None
	GCGCCTGTGAGCAGCtGgaGAGG	14	54348869	+	3	45 kb upstream of *CDKN3* TSS; overlaps CTCF TF binding site for *CDKN3*; in DNase I hypersensitive region; in region of H3K4me1 enrichment	*CDKN3*

Supplementary Table 3. shRNA recognition sequences for *PTEN* repression. Numbering refers to start position relative to the TSS of *PTEN* mRNA transcript variant 1 (Genbank accession number NM_000314.8) in mRNA not DNA. nt: nucleotide.

shRNA	Recognition sequence	Location in *PTEN* transcript
+874	GCAGAAACAAAAGGAGATATCA	Exon 1
+1436	GATGATGTTTGAAACTATTCCA	Exon 5
+1608	GTAGAGTTCTTCCACAAACAGA	Exon 6
+2022	GATGAAGATCAGCATTCACAAA	Exon 9 (1 nt mismatch)

Supplementary Table 4. TaqMan Gene Expression Assays used for qRT-PCR in *PTEN* repression experiments. For each gene, the TaqMan Assay ID, targeted RefSeq transcripts and reporter dye are listed.

Gene	Assay ID	RefSeq sequence(s)	Dye
PTEN	Hs02621230_s1	NM_000314.8<br>NM_001304717.2<br>NM_001304718.1	FAM-MGB
GAPDH	Hs02786624_g1	NM_001256799.2<br>NM_001289745.1<br>NM_001289746.1<br>NM_002046.5	FAM-MGB
SAMD11	Hs00942141_m1	NM_152486.2	FAM-MGB
KLF16	Hs00259103_m1	NM_031918.3	FAM-MGB

Supplementary Table 5. Primers used for SYBR Green qRT-PCR in *PTEN* repression experiments.

Gene	Forward primer	Reverse primer
COX17	GCTCATAGCTGCTTTTGGCG	TCACACAGCAGACCACCATT
FOXD1	ATTGAACCCGAGAACGTCCG	TCAGATGCGTGCGTTACAGA
VPS9D1	CGCCATGAAGCTTGCCAAC	ATGCTCCTCAGGTATTCCGT
ZNF276	GGGTCCTCTAGGAACCTCGG	GACGACCCAGGAGACAGGAA
KCNH2	CAGCTCGAGCCAGGCAG	ATGAAGCCAGGGTGGTTGTG
REXO1	TCCAGGACAATGTGGATGGG	GTCTTGGCGTCTTCTCGAAC
MPRIP	CACCTGCCTACGTGGACTC	CCAGGGTGGCATATTGCCTG
CDKN3	AGCCGCCCAGTTCAATACAA	TCACTCGTGACAAAGATAGCCA
GAPDH	CTTAGCACCCCTGGCCAAG	GGCAGGGATGATGTTCTGGA
Pten (rat)	TTGAAGACCATAACCCACCACAG	CACAAATCATTACACCAGTCCGTC
Ppia (rat)	AGCATACAGGTCCTGGCATC	TTCACCTTCCCAAAGACCAC

Supplementary Table 6. Antibodies used for Western blotting in *PTEN* repression experiments. For each antibody, the isotype, manufacturer, catalogue number, molecular weight of the antigen and dilution factor in *PTEN* repression experiments are listed.

Antibody	Isotype	Manufacturer	Cat. no.	Molecular weight	Dilution
PTEN	Rabbit IgG	CST	9559	54 kDa	1/1000
GAPDH	Rabbit IgG	CST	2118	37 kDa	1/5000
Rabbit-HRP	Goat IgG	Jackson ImmunoResearch	111-035-144	—	1/10 000
Mouse-HRP	Goat IgG	Jackson ImmunoResearch	115-035-003	—	1/10 000

Supplementary Table 7. Primers used for ChIP-qPCR of *PTEN* and *GAPDH* regulatory regions.

Gene	Forward primer	Reverse primer	Product size
PTEN	ATGTGGCGGGACTCTTTATG	GCGGCTCAACTCTCAAACTT	119 bp
GAPDH	TACTAGCGGTTTTACGGGCG	GAGGCTGCGGGCTCAATTT	137 bp

Supplementary Table 8. Antibodies used for immunofluorescence in *PTEN* repression experiments. For each antibody, the isotype, manufacturer, catalogue number and dilution factor in *PTEN* repression experiments are listed.

Antibody	Isotype	Manufacturer	Cat. no.	Dilution
GFP	Mouse IgG	Roche	11814460001	1/500
Nestin	Rabbit IgG	Biolegend	841901	1/500
Β-III tubulin	Rabbit IgG	Biolegend	802001	1/500

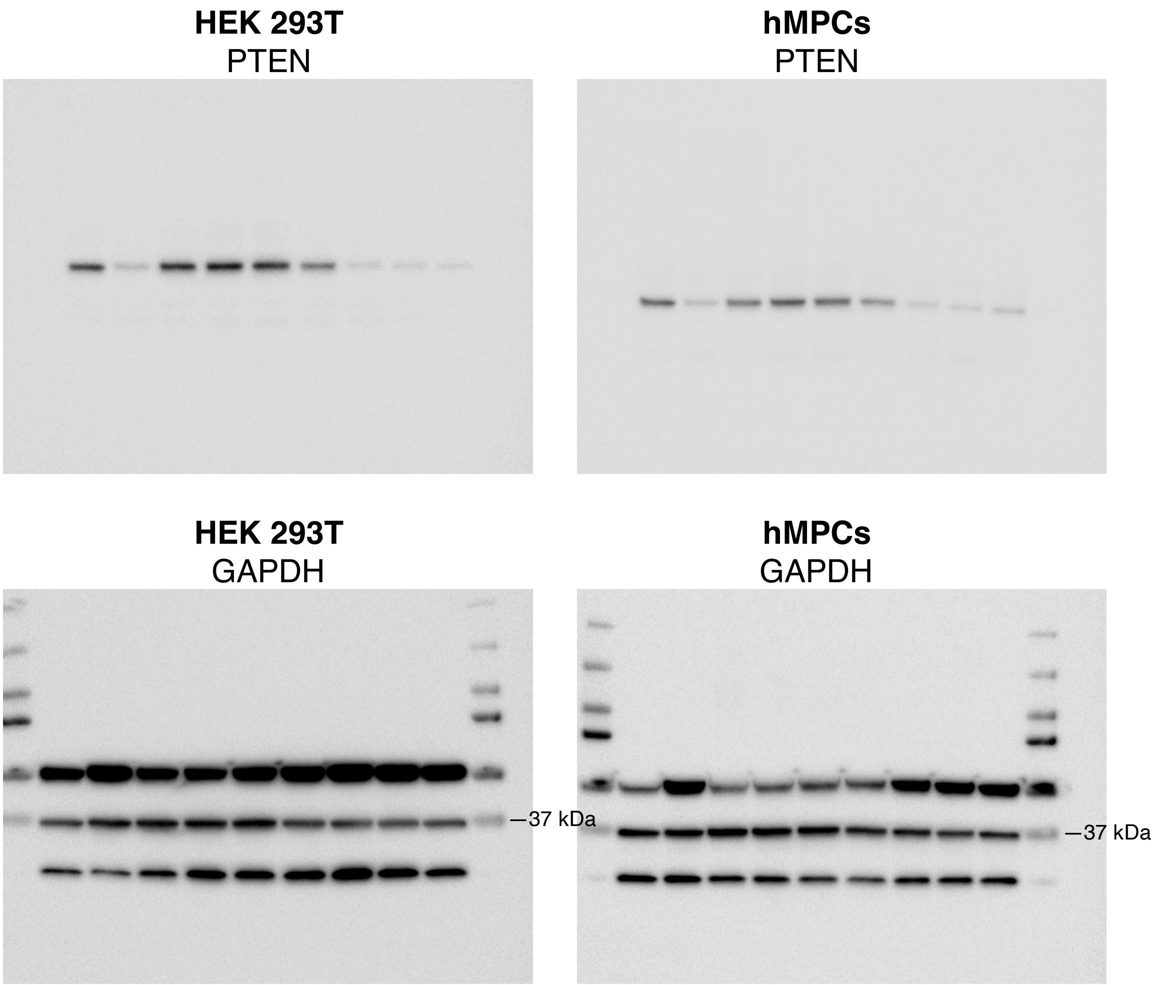

Supplementary Figure 1. Uncropped images of Western blots from Figure 2. GAPDH blots show Bio-Rad Precision Plus Protein Kaleidoscope Prestained Protein Standards with 37 kDa standard annotated.
